# Topological representations of crystalline compounds for the machine-learning prediction of materials properties

**DOI:** 10.1038/s41524-021-00493-w

**Published:** 2021-02-05

**Authors:** Yi Jiang, Dong Chen, Xin Chen, Tangyi Li, Guo-Wei Wei, Feng Pan

**Affiliations:** 1 School of Advanced Materials, Peking University, Shenzhen Graduate School, Shenzhen, PR China.; 2 Department of Mathematics, Michigan State University, East Lansing, MI, USA.

## Abstract

Accurate theoretical predictions of desired properties of materials play an important role in materials research and development. Machine learning (ML) can accelerate the materials design by building a model from input data. For complex datasets, such as those of crystalline compounds, a vital issue is how to construct low-dimensional representations for input crystal structures with chemical insights. In this work, we introduce an algebraic topology-based method, called atom-specific persistent homology (ASPH), as a unique representation of crystal structures. The ASPH can capture both pairwise and many-body interactions and reveal the topology-property relationship of a group of atoms at various scales. Combined with composition-based attributes, ASPH-based ML model provides a highly accurate prediction of the formation energy calculated by density functional theory (DFT). After training with more than 30,000 different structure types and compositions, our model achieves a mean absolute error of 61 meV/atom in cross-validation, which outperforms previous work such as Voronoi tessellations and Coulomb matrix method using the same ML algorithm and datasets. Our results indicate that the proposed topology-based method provides a powerful computational tool for predicting materials properties compared to previous works.

## INTRODUCTION

Advances in materials science are typically slow and arduous^1^, and thus is particularly challenging to meet the increased demand for material characterization^2^. Because the number of possible materials is estimated to be as high as a googol (10^100^)^3^, there is an urgent need for innovative methods and techniques in materials research. To accelerate the development of new materials, high-throughput computing methods have been proposed in recent years, especially the density functional theory (DFT) which can predict the properties of both experimental and hypothetical inorganic compounds^4,5^. The combination of both experiments and computer simulations has proven to be a powerful approach to reduce the time and cost of materials design and has been widely used in Li-ion batteries, electrocatalysis, thermoelectrics, and structural alloys. It has also promoted the establishment of large and high-quality open databases such as Materials Project (MP)^6^, Open Quantum Materials Database (OQMD)^7^, the Automatic Flow of Materials Discovery Library^8^, and MaterialGo^9^. While DFT approaches are powerful, they are inefficient and prohibitively expensive for heavier elements, strongly correlated electrons, and large molecules. In addition, all physical methods, including DFT, are not designed to deal with massive and diverse datasets in materials sciences.

With the development of large databases and improved algorithms, machine learning (ML) has emerged as a great promising approach in the research of inorganic crystal structures and molecules. It offers a revolutionary tool for rapidly estimating the results of DFT calculations or experimental data by creating prediction models from databases. ML algorithms aim to optimize the generalization performance of models. ML might be generally split into three main categories: supervised learning, unsupervised learning, and reinforcement learning. During the process of supervised learning, systems are exposed to large amounts of labeled data to find the unknown function that can extrapolate the result of unlabeled data. In contrast, unsupervised learning tasks with identifying patterns in data and trying to looks for unlabeled data that can be grouped by similarities. Reinforcement learning aims to learn good policies for sequential decision problems by optimizing a cumulative future reward signal^10^. ML has been successfully applied to predict materials properties including formation energy^11–13^, bandgap^14–19^, thermal conductivity^20–22^, and elastic modulus^23–25^. It can be used to create atomic potential^26,27^, screen functional materials^28–30^, and analyze complex reaction networks^31^.

Descriptors or features, as a pivotal ingredient of a ML model, provide a representation of each molecule in a data set^32^. A poor representation that is either unable to reduce the complexity of the data or unable to maintain vital material information will inevitably lead to large prediction errors. More specifically, descriptors in materials science should capture the information that could distinguish between different atomic and crystal environments^33^. Several different strategies have already been proposed to extract the quantitative representations of crystal materials. Potential energy is predicted by the transformation of pairwise distance models^34^, which only work for a fixed number of atoms and are not unique under the permutation of atoms. Several models rely on the dataset of compounds with the same stoichiometry or the same structure^11,35,36^. However, to fully cover highly diverse compositions and structures in crystal materials, a rotational, transnational, and scale-invariant representation is needed to empower ML models. Faber et al.^37^ proposed three generalized Coulomb matrix (CM) approaches. One approach considers full Coulomb interactions between two atoms in a lattice setting. The second one models atomic electrostatic interactions in the unit cell and its nearest neighbor environment. In the last approach, they replace the Coulomb interaction by a periodicity potential with respect to the lattice vectors. Their approaches achieved 0.37 eV/atom mean absolute error (MAE) in predicting the formation energies of new structures. Schütt et al.^38^ constructed a ML model to predict the density of states at the Fermi energy based on the crystal representation called partial radial distribution function which is invariant under translation, rotation, and the choice of the unit cell. Ward et al.^39^ applied the standard random forest (RF) to predict the formation energy based on features derived from Voronoi tessellations to represent structural properties and atomic properties. This model achieves an MAE of 80 meV/atom in cross-validation for a dataset of 435,000 formation energies. These feature engineering-based ML models with structural descriptors have achieved exceptional accuracy prediction. Xie et al.^40^ proposed another idea to construct features by crystal graph convolutional neural network, which is invariant for unit cell choices and achieves a high prediction accuracy of DFT calculations on many properties. However, neural networks are well-known “black box” and involve too many parameters. After intensive training, the final predictors are hard to interpret physically. In contrast, topology considers the global connectivity of various components in a space and studies isolated entities, rings, and higher dimensional faces within the space^41^. It turns out that traditional topology gives rise too much geometric reduction to provide useful description of crystal structures^42^. Persistent homology, however, is able to bridge geometrical shape analysis and topological characterization by embedding multiscale geometric information into topological invariants^43^. It generates a nested family of topological spaces by varying a filtration parameter, which results in topological invariants of various dimensions, namely, isolated components, circles, and cavities, corresponding to Betti-0, Betti-1, and Betti-2, respectively. Molecule-level persistent homology neglects chemical and biological properties, element-specific persistent homology has been proposed to retain crucial biological information^44–46^. This method has been applied to represent organic molecular and biomolecular properties^44–46^. The successful application in biomolecules motivates us to utilize persistent homology to represent crystal compounds for predicting their physical properties.

In this work, we propose a enhanced approach for predicting properties of crystalline materials using a topological representation derived from persistent homology. The direct application of persistent homology without considering atomic diversity and crystal periodicity is not suitable for crystal property predictions. Therefore, we introduce atom-specific persistent homology (ASPH) to extract atom-specific crystal information for representing crystal structures in ML. ASPH offers a variety of atom-specific topological fingerprints in the crystal cell and adapts this representation to periodic systems. In this work, we employ a large dataset from Inorganic Crystal Structure Database (ICSD)^47^ and OQMD to compare our proposed method against existing methods in the literature (i.e., Voronoi tessellations and CM) via cross-validation. Combined with composition-based attributes, our method achieved excellent results with the mean absolute error as low as 61 meV/atom. Moreover, to understand the limitations of our topology-based ML method, we analyze the outliers with large errors in our predictions with respect to DFT calculations. We explore the types of compounds that are more likely to cause large deviations in our predictions.

## RESULTS AND DISCUSSION

### General performance

**Fig. 1:**
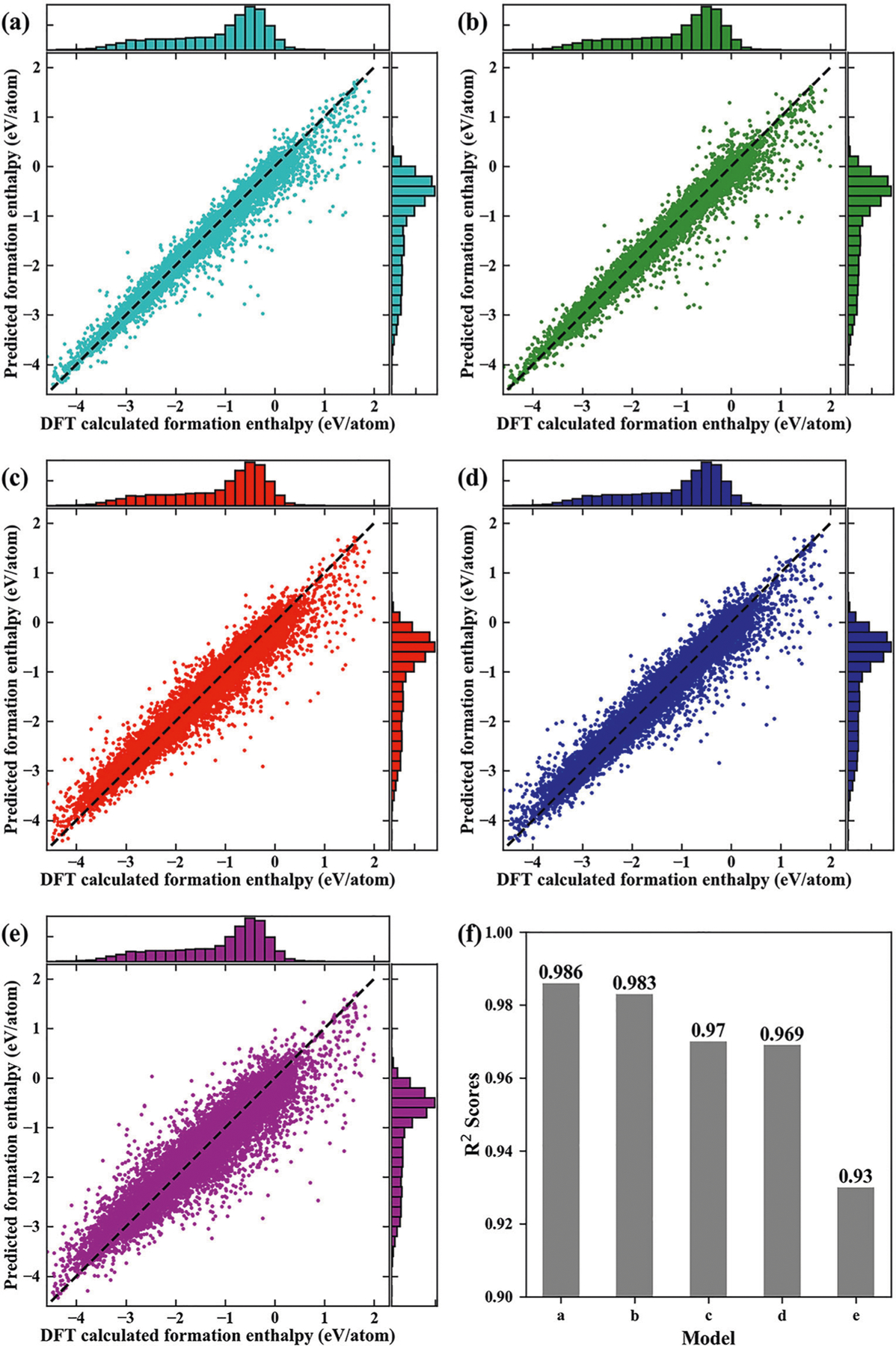
Comparison of DFT-calculated formation enthalpy and predicted formation enthalpy (eV/atom) for different methods. **a** ASPH + Comp, **b** Voronoi tessellations, **c** ASPH, **d** Betti-0 ASPH, and **e** CM-sine. The top and right subfigure is the distribution of calculated data from ICSD and predicted data, respectively. **f** *R*^2^ results for different models. All prediction data are the average performance obtained from tenfold cross-validations with 20 repetitions.

**Fig. 2:**
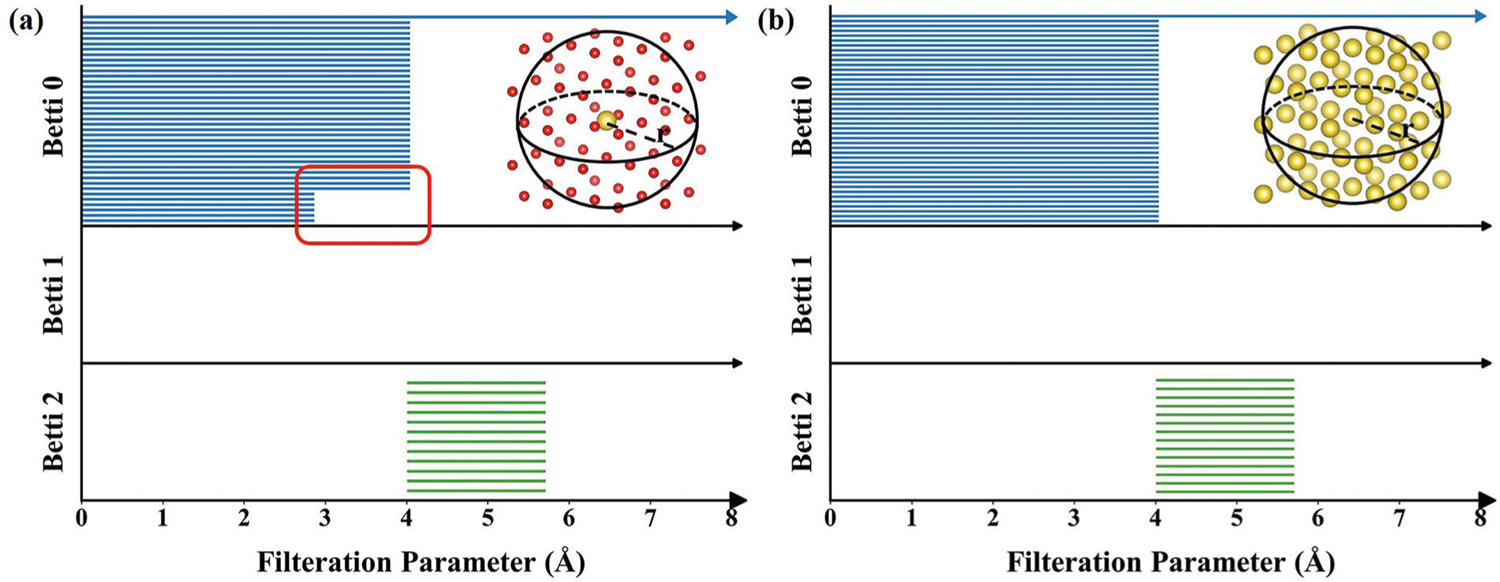
Illustration of persistence barcodes of NaCl crystal specific central-atom persistent homology. The central atom is Na with the surrounding atom is Cl (**a**) and is Na (**b**), respectively. Charts from top to bottom are Betti-0, Betti-1, and Betti-2 barcodes, respectively. The point cloud of each barcode is displayed.

In Table 1, we compare the performance of the proposed topology-based method with those of Voronoi tessellations^39^ and Coulomb Matrix-based method in the literature^37^ using the same machine learning algorithm and the same set of hyperparameters. The scatter plots of the predictions and performance for different prediction methods are shown in Fig. 1. ASPH refers to the method using topological invariants Betti-0, Betti-1, and Betti-2. Similarly, Betti-0 ASPH only use Betti-0 for feature generations. The coefficients of determination (*R*^2^), the root mean squared error (RMSE), and the mean absolute error (MAE) for tenfold cross-validations repeated 20 times are given for various methods. An MAE of 61 meV/atom is achieved by ASPH combined with composition-based features, whose performance is better than those of Voronoi tessellations and CM modified by sine matrix approximation. Our mean prediction error is also lower than the error of DFT approximation to experimentally measured formation enthalpies. Moreover, we find that the MAE from Betti-0 ASPH is slightly larger than the one from ASPH. We found that it is necessary to add topological invariants Betti-1 and Betti-2 to feature because they can capture the many-body interactions and reflect the symmetry of crystal structure. To better illustrate our findings, we consider a highly symmetrical structure NaCl in Fig. 2. With the radius of filtration increasing, when there is only one component, which means all atoms in neighbor are in connection, all 1-simplex in the point cloud directly turn into 3-simplex. Therefore, there is no Betti-1 in its point cloud. In addition to that, we find that if using only Betti-0 representations, we can also achieve good performance. Apart from having a lower MAE, the model created by our method has a better result on *R*^2^ and RMSE as well. Overall, for our method, predicted values of 28195 (88.3%) materials are within 25% of the DFT-calculated values, and only 53 (0.17%) predicted values have errors over 1 eV/atom. In contrast, for Voronoi tessellations and CM-sine, 27,948 and 22,404 predicted values are within 25% accuracy of computed values while 66 and 315 predicted values have errors over 1 eV/atom, respectively. Supplementary Fig. S1 shows the learning curves of multiple model prediction performance with respect to the training size. It indicates that topological attributes provide important information about the crystal materials and improve accuracy compared with composition-only features. Moreover, our model prediction accuracy is higher than other methods as the amount of the training data increases. Because the topological properties provide unique information when there are multiple structures with the same composition.

### Systematic errors analysis

**Fig. 3:**
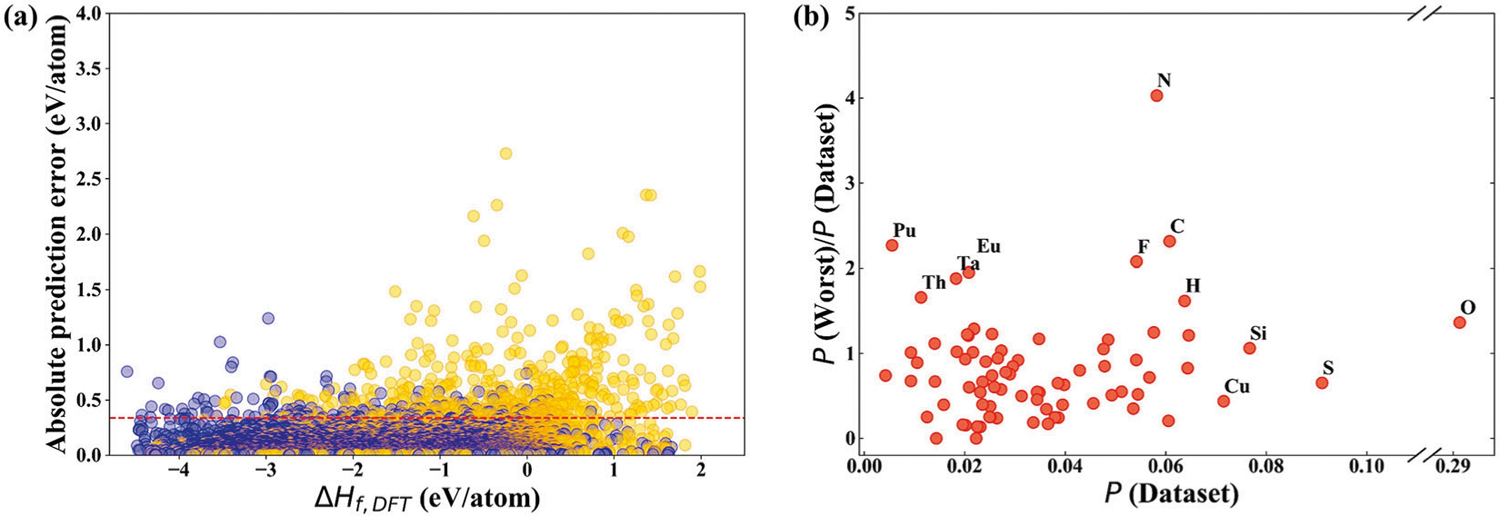
Analysis of ML model prediction error. **a** An illustration of the DFT-calculated formation enthalpies of various compounds and their absolute prediction errors in 10-fold cross-validations. The red dashed line refers to the error value to which 98% of absolute prediction errors are smaller or equal. The yellow dots indicate that the ML predicted values are less than those of the DFT while the blue dots are the opposite. **b** An illustration of the occurrence probabilities of various elements in the whole dataset *P*(Dataset) vs. the ratios of their occurrence probabilities in the set of 2% highest prediction errors [*P*(Worst)] over *P*(Dataset).

To evaluate the scalability of our topological based model, we analyze the absolute prediction error in the tenfold cross-validations of our method for each compound in the dataset. We select the 638 compounds with the highest 2% prediction error values (i.e., above 0.336 eV/atom) to understand the set of compounds that are difficult to be accurately predicted. From Fig. 3a, it is clear that many of these compounds have positive formation enthalpies, suggesting they are thermally unstable. Therefore, the unstable compounds are most likely outliers and their experimental values are subject to large errors. It is likely true that the original DFT calculations were also unreliable for these compound.

We also found that compounds that contain elements that occur less frequently in our dataset are more likely to appear in the set with higher prediction errors. Figure 3b shows the comparison of the probability of finding a given compound with a specific element in dataset [*P*(Dataset)] and the ratio between the probability of finding that element in the set of 2% highest prediction errors [*P*(Worst)] and the probability of finding the same element in our entire dataset. The least frequently occurring elements in our dataset such as Pu, Re, Ta, and Eu, have a higher probability to appear in the set with top 2% highest prediction errors. From the above results, it is clear that our model is less predictive for molecules having rarely occurring elements in the database. This is true for ML-based methods in general.

There are three elements, N, C, and F, that occur frequently in the entire dataset but are some of the worst predicted compounds. In 638 compounds with high prediction errors, there are 217 compounds that contain N, C or F. We found that these elements occur in association with other rarely-occurring elements, such as IrN, TlF_3_, IrC_4_, which makes their accurate predictions very difficult. Therefore, the [*P*(Worst)] of these three elements will also be relatively high. Additionally, a few compounds with element F have positive formation enthalpies (3/1730) while other compounds contain element F have negative formation enthalpies. Therefore, the predicted formation enthalpies of these three outlier compounds with element F are negative and their absolute prediction errors are larger than 1 eV/atom.

AlPO_4_ (ICSD #162670) structure shown in Supplementary Fig. S2 exhibits the worst prediction accuracy with an absolute prediction error (APE) of 2.73 eV/atom. Compared with other stereoisomerisms in our dataset, we find that most of DFT-calculated formation enthalpy values are <−2.90 eV/atom while there are two outliers with Δ*H*_*f*_ = −0.24 eV/atom, −0.61 eV/atom and APE = 2.73 eV/atom, 2.16 eV/atom, respectively. An illustration of DFT-calculated enthalpy values and the APE of AlPO_4_ is shown in Supplementary Fig. S3. For similar reasons, BaTiO_3_ (ICSD #109327), BN (ICSD #27986), CaO (ICSD #261847), VS_2_ (ICSD #68713), etc. also have high errors. From our analysis of their failures, we conclude that our model does not give accurate predictions of formation enthalpy values for stereoisomerisms having a diverse formation enthalpy distribution. A possible reason is that the procedure of processing topological information is oversimplified so that ML algorithm does not do a good job in differentiating stereoisomerisms.

Contrary to geometry that widely used in crystal structure descriptors, topology is rarely implemented in quantitative analysis of materials science. In this work, we propose atom-specific persistent homology (ASPH) and apply it to material science analysis via machine learning (ML) models. Unlike high-level abstraction of conventional topology, the proposed ASPH embeds multiscale geometric information into topological invariants with chemical insights. It can effectively extract unique features such as independent components, loops, and cavities. More specifically, independent components are associated bond lengths of pairwise interactions, while loops and cavities capture many-body interactions.

Furthermore, our ASPH can deal with crystalline compounds which have structural periodicity and elemental diversity. Extensive experiment shows that our model provides a reliable estimation of DFT calculations using around 30,000 training data with diverse structural types and compositions. Moreover, it offers a more accurate prediction in cross-validations than previous methods do^37,39^. Its applicability extends to all space groups and a great majority of elements. Finally, the success of this method enables us to discover new materials with desirable properties significantly faster and cheaper.

## METHODS

### Simplex and simplicial complex

**Fig. 4:**
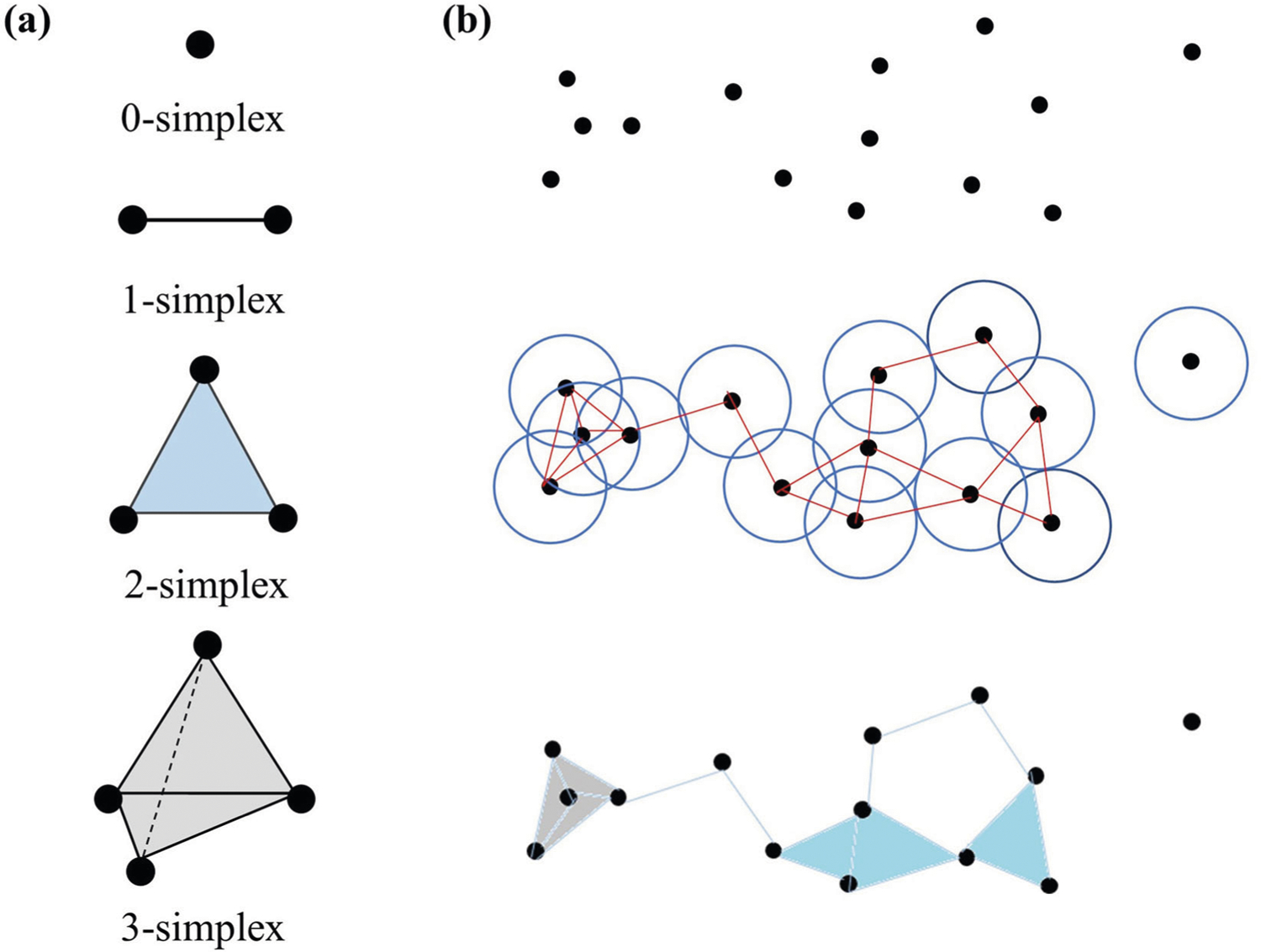
Basic simplexes and simplicial complex construction in a given radius of filtration. **a** From top to bottom an example of a 0-simplex, 1-simplex, 2-simplex, and 3-simplex. **b** The construction of simplicial complex. There are one 0-simplex, six 1-simplexes, two 2-simplexes and one 3-simplex.

Topological data analysis uses a simplices and simplicial complexes for the description of complex shapes, which are mathematically and computationally easier to process than their original counterparts. A set of *k* + 1 affinely independent points in ${\mathbb{R}}^{k}$ is a *k*-simplex denoted by *σ*^*k*^ which can be represented by $\left\{v_{0},v_{1},\cdots ,v_{k}\right\}$ and each *v*_*i*_ is called a vertex of the simplex. Specifically, a 0-simplex is a vertex, a 1-simplex is a line segment, a 2-simplex is a triangle and a 3-simplex is a tetrahedron, as shown in Fig. 4. A subset of the *k* + 1 vertices of a *k*-simplex with *m* + 1 vertices forms a convex hull in a lower dimension and is called an *m*-face of the *k*-simplex. An *m*-face is proper if *m* < *k*. A simplicial complex is a set of simplices which are convex hulls of affinely independent points. More specifically, a simplicial complex is a finite collection of simplices $X={\left\{\sigma_{i}\right\}}_{i}$ satisfying that the intersection of any two simplices in *X* is either empty set or a common face of the two and all the faces of a simplex in *X* is also in *X*. The collection of all *k*-simplices in *X* is denoted *X*_*k*_. The dimension of a simplicial complex is the highest dimension of its simplices.

### Homology

For a simplicial complex *K*, a *k*-chain *c*_*k*_ of *K* is the sum of the form of *k*-simplices in *K*, and *k* is not greater than dimension of *K*, and is defined as $c_{k}=\Sigma \alpha_{i}\sigma_{i}$ where *σ*_*i*_ is the *k*-simplices and *α*_*i*_ is coefficients. Generally, *α*_*i*_ can be set as elements of a field such as ℝ, ℚ, or ${\mathbb{Z}}_{n}$. For simplicity, it is commonly chosen to be ${\mathbb{Z}}_{2}$. The group of *k*-chains in *K*, denoted *C*_*k*_, with operation of modulo 2 addition can form an Abelian group $\left(C_{k},{\mathbb{Z}}_{2}\right)$. So we can extend the definition of the boundary operator to chains, showed in Eq. (1).

The boundary operator applied to a *k*-chain *c*_*k*_ is defined as

(1) $$\partial_{k}\sigma_{k}=\sum \alpha_{i}\partial_{k}\sigma_{i},$$

where *σ*_*i*_’s are *k*-simplices. The boundary operator is a map from *C*_*k*_ to *C*_*k*−1_, which is also named boundary map for chains. Note that operator ∂_*k*_ satisfies the property that $\partial_{k}\partial_{k+1}=Ø$ for any (*k* + 1)-simplex *σ* following the fact that any (*k* − 1)-face of *σ* is contained in exactly two *k*-faces of *σ*. The chain complex is defined as a sequence of chains connected by boundary maps with decreasing dimensions and is represented as

(2) $$\cdots \to C_{n}\left(K\right)\overset{\partial_{n}}{\to }C_{n-1}\left(K\right)\overset{\partial_{n-1}}{\to }\cdots \overset{\partial_{1}}{\to }C_{0}\left(K\right)\overset{\partial_{0}}{\to }0.$$

In other words, through the application of two boundary operations, the *k*-chain is mapped to an empty set $\partial_{k}\partial_{k+1}=Ø$, we can define *k*-cycle group and *k*-boundary group which are the subgroups of *C*_*k*_ as kernel and image of ∂_*k*_ and ∂_*k* + 1_, respectively,

(3) $$Z_{k}=\text{Ker}\partial_{k}=\left\{c\in C_{k}|c=Ø\right\},$$

and

(4) $$B_{k}=\text{lm}\partial_{k+1}=\left\{c\in C_{k}|\exists d\in C_{k+1}:c=\partial_{k+1}d\right\}.$$

where *Z*_*k*_ is the *k*-cycle group and *B*_*k*_ is the *k*-boundary group. With the aforementioned definitions, the *k*-homology group is defined to be the quotient group of the *k*-cycle group modulo the *k*-boundary group,

(5) $$H_{k}=Z_{k}/B_{k}.$$

where *Z*_*k*_ is the *k*-homology group. The *k*th Betti number is defined to be rank of the k-homology group as *β*_*k*_ = *rank*(*H*_*k*_).

### Filtration and persistent homology

Original homology is oversimplified for geometric analysis. Persistent homology introduces the nested sequence of subcomplexes to describe inclusive topological space which depends on a filtration parameter. Specifically, the filtration process of a simplicial complex *K* as a nested sequence of subcomplexes of *K*,

(6) $$Ø\subseteq K_{0}\subseteq K_{1}\cdots \subseteq K_{n}=K.$$

Subcomplexes corresponding to various filtration parameters offer the topological fingerprints of multiple scales. The *k*th persistent Betti numbers $\beta_{k}^{i,j}$ are ranks of *k*_*th*_ homology groups of *K*_*i*_ that are alive and are defined as

(7) $$\beta_{k}^{i,j}=\text{rank}\left(H_{k}^{i,j}\right)=\text{rank}\left(Z_{k}\left(K_{i}\right)/\left(B_{k}\left(K_{j}\right)\cap Z_{k}\left(K_{i}\right)\right)\right).$$

These persistent Betti numbers are used as topological fingerprints in machine learning studies of materials. There are different types of simplicial complex constructions used in persistent homology. The Vietoris–Rips (VR) complex used in this work is formed by all points in it has pairwise distances no greater than a cutoff distance *d* in a given metric space. The abstract property of the VR complex enables the construction of simplicial complexes for correlation function-based metric spaces, which models pairwise interaction of atoms with correlation functions instead of spatial metrics.

### Atom-specific persistent homology

Persistent homology only offers the global structural information which cannot represent crystal structures with a wide range of chemical compositions and structural complexity. We introduce atom-specific persistent homology to embed atom-wise chemical information into topological invariants. The essential idea is that, in a unit cell, there are only a few atoms and each atom has its unique structural environment, which defines its own topological fingerprints.

**Fig. 5:**
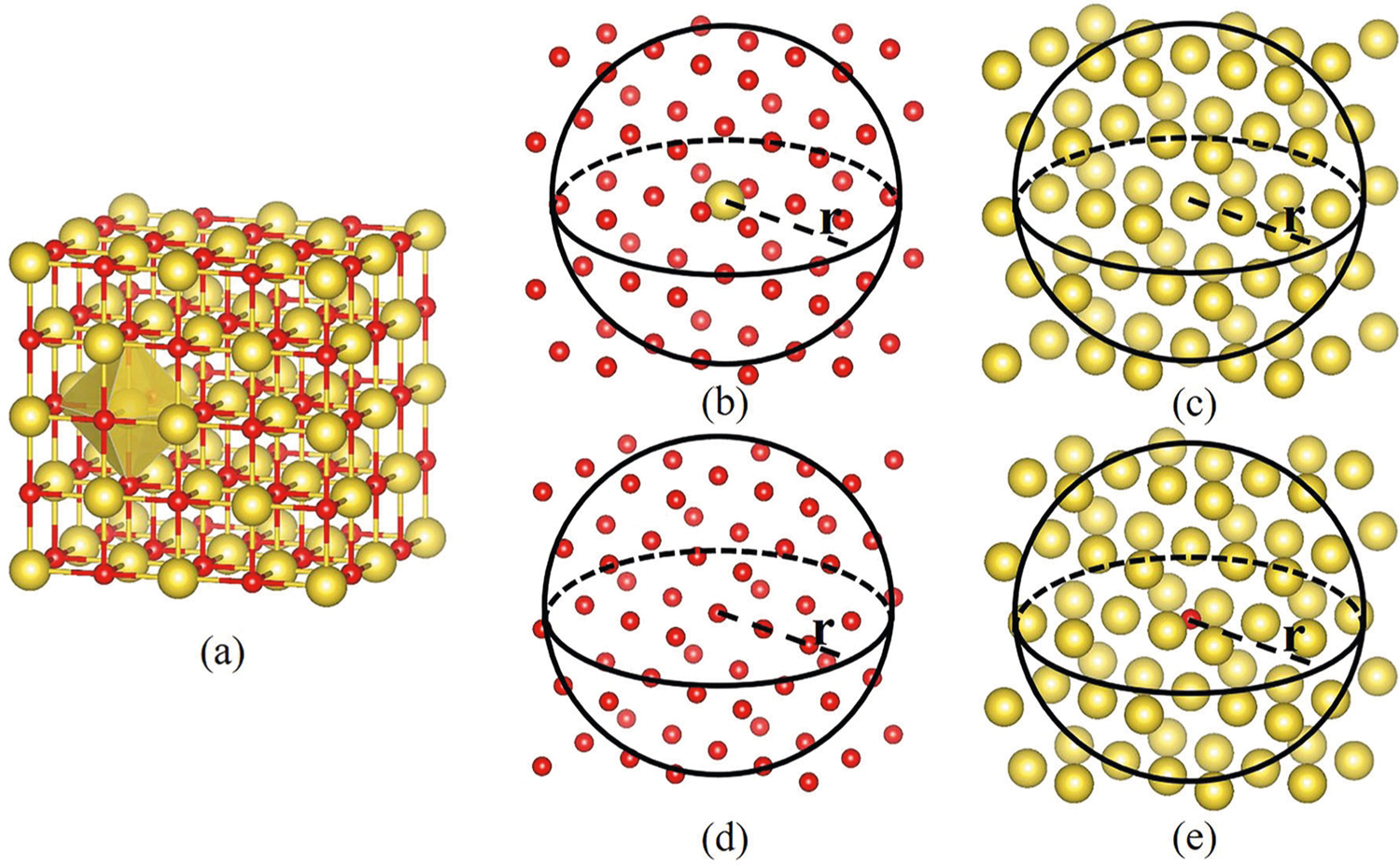
Illustration of atom-specific persistent homology point clouds. **a** the original crystal structure of NaCl with red atom being Cl and yellow atom being Na. Four atom-specific point clouds are established by Na-Cl (**b**), Na-Na (**c**), Cl-Cl (**d**), and Cl-Na (**e**), respectively.

Taking the classic NaCl crystal as an example, one can choose either the Na atom or the Cl atom as the atom of interest to generate atom-specific topological fingerprints. For each choice, there are two types of environments, namely Cl atoms or Na atoms. As a result, we have four possible combinations, namely Na-Na, Na-Cl, Cl-Na, and Cl-Cl. Their atom-specific point clouds are shown in Fig. 5.

In general, to capture element-level interactions, we consider the combination of all element pairs *P* for the substance composition. Given a specific composition, persistence barcodes are calculated as follows. The element-specific pair $P_{\alpha ,i}^{\beta }$ represents a collection of pairs of atoms around the *i*th central atom of element type *α* and surrounding atoms of element type *β*, where *α* and *β* may be the same. First, expanding the unit cell so that the distance between the boundary atoms and any atoms in the original unit cell is smaller than the pre-defined cutoff radius *r*_*c*_. Then, for the *i*th atom of interest in the unit cell, a point cloud consisting of all atoms within a cutoff radius *r*_*c*_ is selected

(8) $$R_{i}^{\alpha ,\beta }\left(r_{c}\right)=\left\{{\text{r}}_{j}^{\beta }|\left\|{\text{r}}_{i}^{\alpha }-{\text{r}}_{j}^{\beta }\right\|<r_{c},{\text{r}}_{j}^{\beta },{\text{r}}_{i}^{\alpha }\in P_{\alpha ,i}^{\beta },\forall j\in 1,2,\cdots ,N\right\},$$

where *N* is the number of atoms in pair $P_{\alpha ,i}^{\beta }$. Given a point cloud, simplicial complex, homology group, and persistence barcode are computed via persistent homology. We compute the persistence barcodes by using software package Ripser^48^. The persistence barcode pair of central atom Na point cloud (Fig. 5b, c) is illustrated in Fig. 2. In the Betti-0 section of Na-Cl barcode, the six bars ended at 2.84 Å indicate that there are six nearest neighbor atoms Cl around central atom Na. The other bars ended at 4 Å show that the distance between any other two nearest neighbor atoms is 4 Å. There is no Betti-1 in this case because the distances between any two components are the same, which reflects the high-level symmetry of the structure.

### Topological representations

**Fig. 6:**
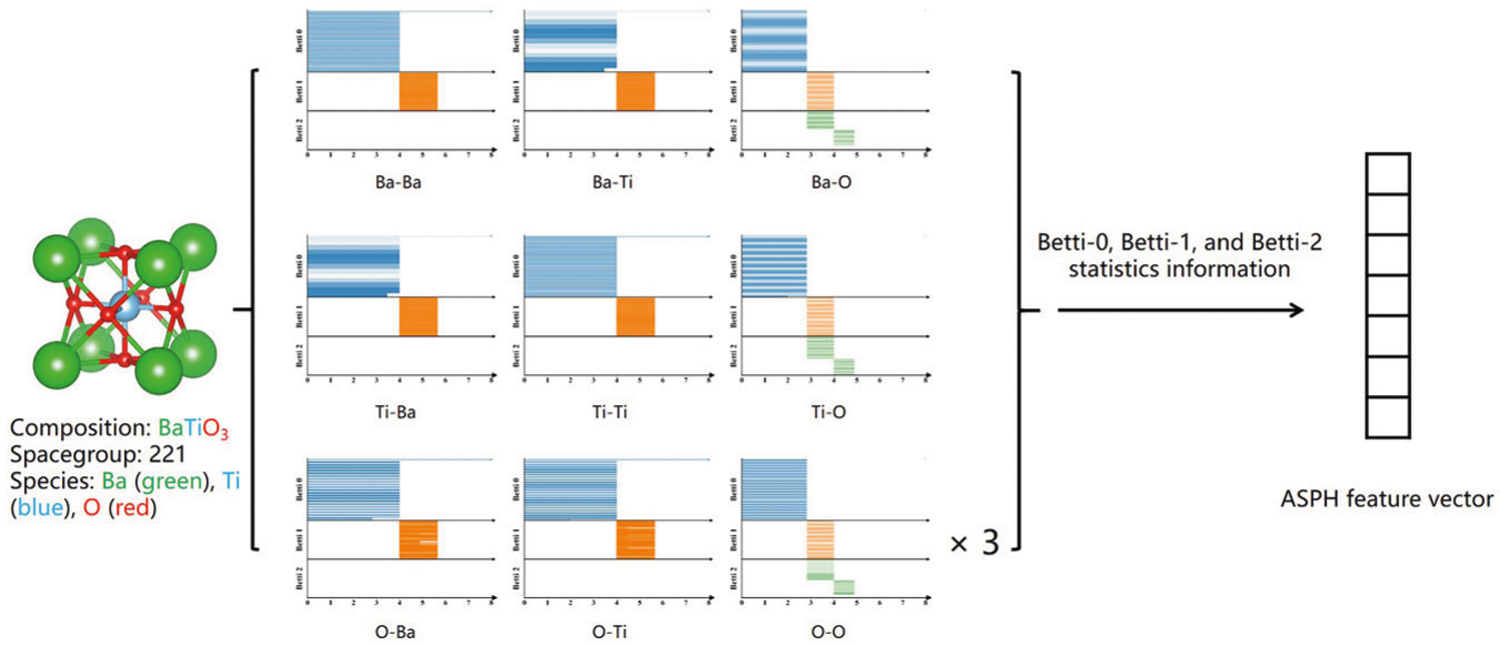
The construction process of BaTiO_3_ topological feature. First, compute all barcodes of the combinations formed by each atom in the unit cell as a central atom and surrounded by various element types. The ASPH features are then generated from the statistics data of these barcodes. Since the unit cell of BaTiO_3_ having three O atoms, the barcode data of this type in the figure is multiplied by three.

The topological representations used in the machine-learning algorithms are extracted from persistence barcodes computed by atom-specific persistent homology. The cutoff radius *r*_*c*_ used to generate the barcodes in this paper is 12 Å. We describe the procedure for generating topological representations for crystalline compounds. The first step is to generate a collection of atom-specific barcodes denoted by $\left\{B\left(P_{\alpha ,i}^{\beta },D\right)\right\}$, where $P_{\alpha ,i}^{\beta }$ was defined above, *i* goes through all atoms in the unit cell, *α* and *β* run over all possible element types, and *D* denotes topological dimensions, such as Betti-0, Betti-1, and Betti-2. Taking BaTiO_3_ as an example, we will have Betti-0, Betti-1, and Betti-2 barcodes for each of five atoms in the unit cell. The second step is to generate a collection of element-specific barcodes denoted by $\left\{B\left(P_{\alpha }^{\beta },D\right)\right\}$. This is done by combining together atom-specific barcodes according to their element types. Using BaTiO_3_ as an example, we will have Betti-0, Betti-1, and Betti-2 barcodes for each of three element types. The third step is to characterize barcodes. In general, for any bar in one barcode, it is important to keep track of its birth, the death, and the persistent length, because this information is related to the bond length, ring, or cavity size. However, for Betti-0 bars, since their birth positions are uniformly 0, only the length of the bar needs to be recorded. The last step is to obtain statistics for each element type of barcodes. Therefore, for the element-specific barcodes of Betti-0 in $B\left(P_{\alpha }^{\beta },D\right)$, five statistical quantities are calculated as the minimum, maximum, average, standard error, and the sum of the bar length. Moreover, for element-specific barcodes of Betti-1 or Betti-2, we generate five statistical quantities, i.e., the minimum, maximum, average, standard deviation, and the sum for each of the birth, death, and persistent length. Therefore, we have a total of 35 element-specific topological representations for each element type. Additionally, we combine all atom-specific barcodes in the unit cell, which leads to another 35 statistical representations. For BaTiO_3_, we (3 + 1) × 35 (140) non-zero representations for BaTiO_3_. Since there are 80 possible element types in the entire dataset, our total number of features is 2835 (i.e., 35 × 81). We set all representations to 0 for element types that do not exist in the molecule of interest. The overall process of element-specific representation generation is shown in Fig. 6. Basically, ASPH are translational, and rotational invariant by design and is able to reflect smooth changes due to perturbations in atomic positions (see Supplementary Figs. S3 and S4).

In addition to topological information described by ASPH, composition-based features are used in our method. These attributes are described in work by Ward et al.^49^. It contains the stoichiometric attributes for the fractions of element, elemental-property attributes based on statistics of the elemental properties of all atoms in the crystal, electronic structure attributes which are the average fraction of electrons from the *s*, *p*, *d* and *f *valence shells between all present elements^50^, and ionic compound attributes consist of differences in electronegativities between constituent elements and whether it is possible to form an ionic compound if all elements in common oxidation states.

### Machine learning algorithm, dataset, and validation

The ASPH and composition features are used as machine learning features to predict inorganic periodic solids formation enthalpies. For ML algorithm selection, we choose to use gradient boosted regression trees (GBRT)^51^ to test the accuracy, robustness, and efficiency of topological based features. GBRT is able to combine a number of weak predictors to create a strong model. The training of a GBRT model is done by adding one tree at a time to reduce the lose function of the current model. In practice, different randomly selected subsets of the training data and features are used for each update of the model to reduce overfitting. Hyper-parameter searching is done by the cross-validation judged by *R*^2^. The hyper-parameters used in GBRT are: n_estimators = 300,000, learning_rate = 0.001, max_depth = 7, min_samples_split = 5, subsample = 0.85 and max_features = sqrt. The ML models are built using scikit-learn software (version 0.19.2)^52^. Our dataset includes 31912 compounds which primitive cell size smaller than 40 atoms, covering the seven lattice systems and 80 elements (H-Pu, excluding noble gases, Tc, Pa, Pm, Po, At, Rn, Fr, Ra, and Ac). Tenfold cross-validation of the data sets is used to verify the proposed method. To address the robustness of the machine learning model, the random splitting of data in tenfold cross-validation is repeated 20 times. The median performance and the standard deviation of the performance across repeated experiments are reported. The replication of Voronoi tessellations and Coulomb Matrix are using Magpie, which is freely available under an open-source license^49^.

## Supplementary Material

Supporting information

## Figures and Tables

**Table 1.** The overall performance of formation enthalpy predictor with different feature extraction method on cross-validation (CV) tasks.

Method^a^	*R* ^2^	RMSE (eV/atom)	MAE (eV/atom)
ASPH + Comp^b^	0.986 (0.000)	0.119 (0.000)	0.061 (0.000)
VT	0.983 (0.000)	0.129 (0.000)	0.067 (0.000)
ASPH	0.970 (0.000)	0.174 (0.000)	0.103 (0.000)
Betti-0ASPH	0.969 (0.000)	0.177 (0.000)	0.108 (0.000)
CM-sine	0.930 (0.000)	0.169 (0.000)	0.267 (0.000)

a The average performance is reported and standard deviations are given in square brackets.

b Comp refers to composition-based attributes.

## Data Availability

The experimental data in this work is in the same Github repository as the code. The structure data is obtained from ICSD^47^, and the DFT-calculated property data is from OQMD^7^.
