# Supplementary material for: Topological representations of crystalline compounds for the machine-learning prediction of materials properties
Source: NPJ Comput Mater. Author manuscript; Available in PMC 2021 Oct 20. (PMC8528346; doi:10.1038/s41524-021-00493-w)
Supplement: Supporting information [file NIHMS1698930-supplement-Supporting_information.pdf]

Supplementary Information

Yi Jiang[1], Dong Chen[1], Xin Chen[1], Guo-Wei Wei[*2] and Feng Pan[*1]

[1] *School of Advanced Materials, Peking University, Shenzhen Graduate School, Shenzhen 518055, People's Republic of China*
[2] *Department of Mathematics, Michigan State University, MI 48824, USA*

Rotational and translational invariance As shown in Fig. S1, the feature of BN (b) is exactly the same as the rotated ones (d). Similarly, Fig. S2 shows our representation is translationally invariant and indicates smooth variations with respect to small changes in the atomic positions.

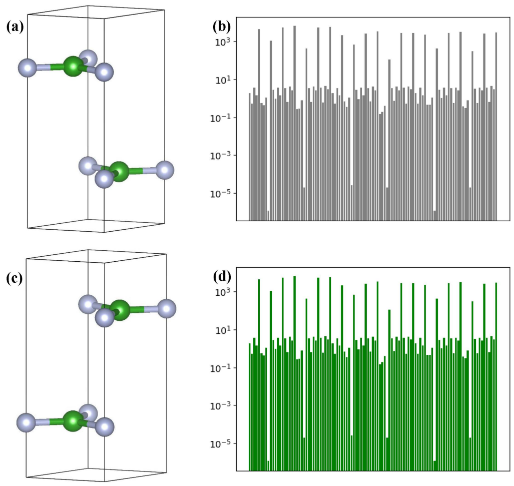

Figure 1: The unit cell of BN and its rotated one, and their respective feature.

*Corresponding author: weig@msu.edu, panfeng@pkusz.edu.cn

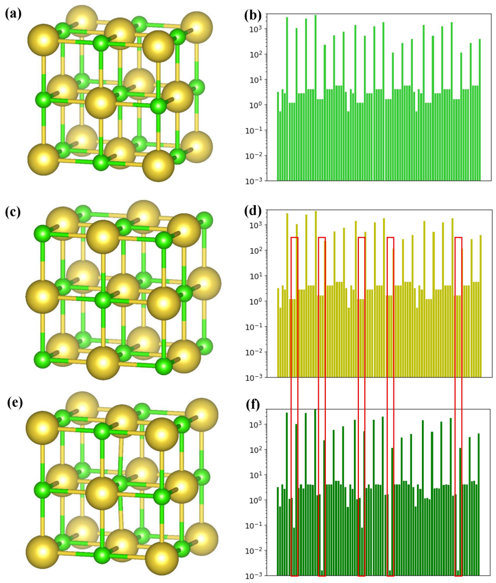

Figure 2: The unit cell of original NaCl, translated NaCl and ones with tiny atomic position change, and their features, respectively. The red frames indicates the changed features.

Learning curves with respect to the training sizes Fig. S3 shows the learning performance of various methods under different training samples. Comp means the compositional feature described by Ward [4]. Comparing with the results of compostion-only attributes and all-attributes, it indicates topology information improves the prediction accuracy of the model as it carries useful information about a material. Moreover, our model prediction accuracy becomes better than other methods as the amount of the training data increases. Because the topological properties provide better information when there are multiple structures with the same composition.

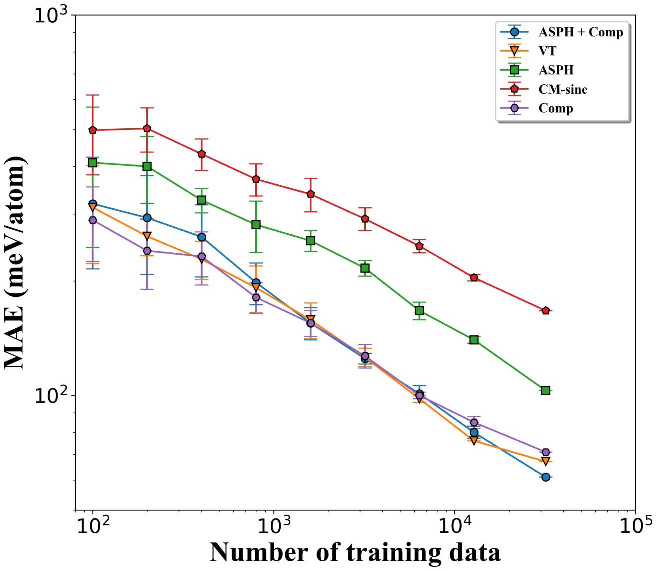

Figure 3: The prediction performance of multiple representations with respect to the training samples.

Unusual Structure The structure of $AlPO_4$ associated with the worst prediction [1] is shown in Fig. S4. Its formation enthalpy is -0.24 eV/atom, which is larger than those of others with same composition (Fig. S5). It behaves abnormally with a relatively short O-O bond of 1.52 Å and the coordination number of the P atom is zero.

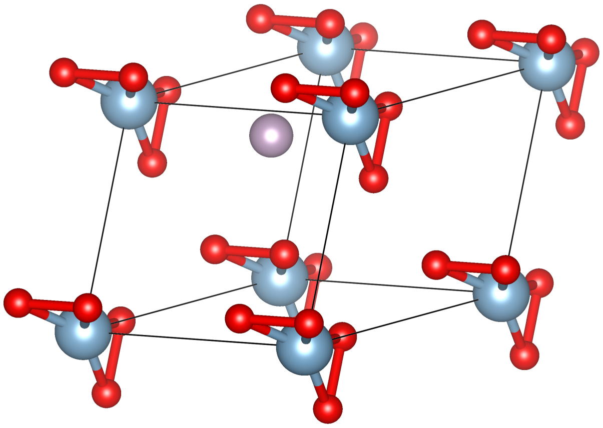

Figure 4: The crystal structure of $AlPO_4$ (ICSD #162670) associated with the worst prediction.

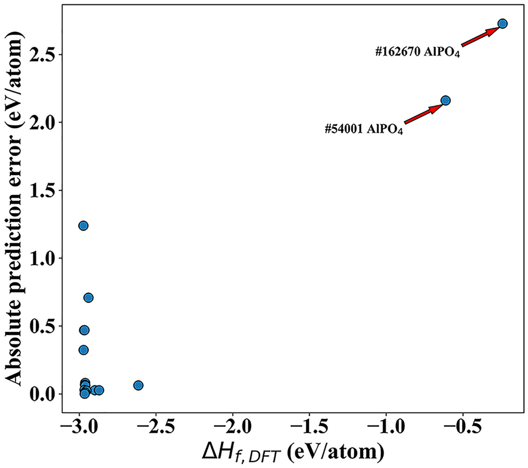

Figure 5: Illustration of DFT-calculated formation enthalpies and the associated absolute errors in our predictions for some forstereoisomerisms of $AlPO_4$.

Software Example script files and the datasets used in this study are included in Github: https://github.com/PKUsamPHTeam/ASPH-Code. The replication of Voronoi tessellations [2] and Coulomb Matrix [3] are using Magpie, which is freely available under an open-source license [4].

References

[1] AF Wright and AJ Leadbetter. The structures of the β-cristobalite phases of SiO_2 and $AlPO_4$. *Philosophical Magazine*, 31(6):1391–1401, 1975.

[2] Logan Ward, Ruoqian Liu, Amar Krishna, Vinay I Hegde, Ankit Agrawal, Alok Choudhary, and Chris Wolverton. Including crystal structure attributes in machine learning models of formation energies via voronoi tessellations. *Physical Review B*, 96(2):024104, 2017.

[3] Felix Faber, Alexander Lindmaa, O Anatole von Lilienfeld, and Rickard Armiento. Crystal structure representations for machine learning models of formation energies. *International Journal of Quantum Chemistry*, 115(16):1094–1101, 2015.

[4] Logan Ward, Ankit Agrawal, Alok Choudhary, and Christopher Wolverton. A general-purpose machine learning framework for predicting properties of inorganic materials. *npj Computational Materials*, 2:16028, 2016.
